# Teaching experimental design: outputs from the FELASA Working Group

**DOI:** 10.1177/00236772241295308

**Published:** 2025-06-09

**Authors:** Derek Fry, Manuel Berdoy, Monica Forni, Carlos Oscar S. Sorzano, Thomas Steckler, Nuno H. Franco

**Affiliations:** 1 School of Biological Sciences, University of Manchester, Oxford Road, Manchester, UK; 2 Biomedical Services, Oxford University, Mansfield Road, Oxford, UK; 3 Department of Medical and Surgical Sciences, 9296 University of Bologna, Bologna, Italy; 4 National Center of Biotechnology (CSIC), Madrid, Spain; 5 Janssen Pharmaceutica NV, Beerse, Belgium; 6 i3S – Instituto de Investigação e Inovação em Saúde, Universidade do Porto, Porto, Portugal

**Keywords:** Experimental design, education and training, learning outcomes, FELASA, 3Rs

## Abstract

Good education and training for scientists undertaking animal experiments is important for providing understanding of key issues in experimental design (ED) and for alleviating continuing concerns about the conduct of animal and *in vitro* research studies. We present here outputs from the FELASA Experimental Design Working Group, set up to consider the current provision of ED teaching and how it might be improved and harmonised across the laboratory animal science community. It is hoped that these outputs will provide practical help to ED teachers who wish to enhance the effectiveness of their teaching; they include

• A list of learning outcomes (LOs) that should be achieved by learners, principally aimed at early career researchers;

• An example of an (adaptable) template of how these LOs could be addressed in 16 h (12 h tuition plus breaks), ideally as a 2-day workshop. If circumstances make 12 h tuition impossible to achieve, key LOs for a shorter course are identified;

• Guidance and recommendations for running ED courses, including some ideas for achieving effective learning, the ideal skill set for tutors, some teaching scenarios, and the amount of statistics to have in a basic experimental design course;

• A glossary of relevant terms (in supplemental material);

• A description of how the 2-day course format ran on two trial occasions, with results of informal assessment of participants as well as their feedback, both immediate and a year afterwards, indicating it was effective;

• A programme for a potential 2-day, training-the-trainers style, workshop, describing its key elements and the results of trialling this with a range of ED tutors.

## L’enseignement de la conception expérimentale: rapport du groupe de travail de la FELASA Résumé

Il est important de fournir aux scientifiques qui entreprennent des expériences sur des animaux un enseignement et une formation de qualité pour qu’ils comprennent les questions clés de la conception expérimentale (ED) et pour apaiser les préoccupations persistantes concernant la conduite d’études menées sur les animaux et de recherches *in vitro*. Nous présentons ici les résultats du groupe de travail de conception expérimentale de la FELASA, mis en place pour examiner la fourniture actuelle de l’enseignement en ED et voir s’il pourrait être amélioré et harmonisé au sein de la communauté scientifique utilisant des animaux de laboratoire. Ces résultats devraient fournir une aide pratique aux formateurs enseignant l’ED qui souhaitent améliorer l’efficacité de leur enseignement.

• une liste des résultats d’apprentissage (LO) qui devraient être atteints par les apprenants, principalement destinés aux chercheurs en début de carrière.

• un exemple de modèle (adaptable) de la façon dont ces résultats d’apprentissage pourraient être abordés en 16 heures (12h de cours plus les pauses), idéalement sous la forme d’un atelier de deux jours. Si les circonstances rendent impossible la formation de 12 heures, les résultats d’apprentissage clés pour un cours plus court sont identifiés

• des conseils et recommandations pour l’exécution des cours ED, notamment quelques idées pour atteindre un apprentissage efficace, l’ensemble de compétences idéal pour les formateurs, quelques scénarios d’enseignement, et la quantité de statistiques à avoir dans un cours de conception expérimentale de base

• un glossaire des termes pertinents

• comment le cours de deux jours s’est déroulé lors de deux ateliers d’essai, les résultats de l’évaluation informelle des participants ainsi que leurs commentaires, à la fois immédiatement et un an après, indiquant son efficacité

• un programme pour une formation potentielle de 2 jours, selon un style de formation du formateur, un atelier pratique, ses éléments clés, et les résultats de l’essai avec une gamme de formateurs d’ED.

## Lehre der Versuchsplanung: Ergebnisse der FELASA-Arbeitsgruppe Abstract

Eine gute Aus- und Weiterbildung von Wissenschaftlern, die Tierversuche durchführen, ist wichtig, um das Verständnis für die Schlüsselfragen der Versuchsplanung zu vermitteln und um die anhaltenden Bedenken hinsichtlich der Durchführung von Tier- und *In-vitro-Forschungsstudien* zu reduzieren. Wir stellen hier die Ergebnisse der FELASA-Arbeitsgruppe für Versuchsplanung vor, die eingerichtet wurde, um das derzeitige Lehrangebot für Versuchsplanung zu prüfen und zu untersuchen, wie es in der Versuchstierforschung verbessert und harmonisiert werden könnte. Diese Ergebnisse sollen Versuchsplanungs-Lehrkräften, die die Effektivität ihres Unterrichts verbessern wollen, praktische Hilfe gewähren und bieten

• eine Reihe von Lernzielen, die von den Lernenden erreicht werden sollten und die sich in erster Linie an Nachwuchsforscher richten

• ein Beispiel für eine (anpassbare) Vorlage, wie diese Lernziele in 16 Stunden (12 Stunden Unterricht plus Pausen), idealerweise in einem zweitägigen Workshop, behandelt werden könnten. Für den Fall, dass die Umstände einen 12-stündigen Kurs unmöglich machen, werden die wichtigsten Lernziele für einen kürzeren Kurs identifiziert

• Hinweise und Empfehlungen für die Durchführung von Versuchsplanungs-Kursen, einschließlich einiger Ideen zur Erzielung eines effektiven Lernerfolgs, der idealen Qualifikationen der Tutoren, einiger Unterrichtsszenarien und des Umfangs der Statistik, der in einem Grundkurs über Versuchsplanung enthalten sein sollte

• ein Glossar mit relevanten Begriffen

• einen Überblick über den Ablauf des zweitägigen Kursformats bei zwei Versuchsanlässen, wobei die Ergebnisse der informellen Bewertung der Teilnehmer sowie deren Rückmeldungen sowohl unmittelbar als auch ein Jahr danach nahelegen, dass es effektiv war

• ein Programm für einen potenziellen zweitägigen Workshop im „Train-the-trainer’-Stil, seine Schlüsselelemente und die Ergebnisse der Erprobung dieses Programms mit einer Reihe von Versuchsplanungs-Lehrkräften.

## Enseñanza del diseño experimental: Resultados del grupo de trabajo FELASA Resumen

Una buena educación y formación para los científicos que realizan experimentos con animales es crucial para comprender aspectos clave del diseño experimental (DE) y para mitigar las preocupaciones continuas sobre los estudios de investigación *in vitro* y sobre la conducta de animales. Presentamos aquí los resultados del Grupo de Trabajo de Diseño Experimental de FELASA, establecido para considerar la oferta actual de enseñanza en diseño experimental (DE) y cómo podría mejorarse y armonizarse en toda la comunidad de Ciencias de Animales de Laboratorio. Estos resultados esperan proporcionar ayuda práctica a los profesores de DE que deseen mejorar la eficacia de su enseñanza y son

• una lista de los resultados del aprendizaje que deben alcanzar los alumnos, dirigida principalmente a los investigadores que inician su carrera profesional

• un ejemplo de una plantilla (adaptable) de cómo podrían abordarse estos resultados de aprendizaje en 16 horas (12h lectivas más descansos), idealmente como taller de dos días. Si las circunstancias imposibilitan la enseñanza de 12 horas, se identifican los resultados de aprendizaje clave para un curso más corto

• orientaciones y recomendaciones para impartir cursos de DE, incluidas algunas ideas para lograr un aprendizaje eficaz, el conjunto ideal de aptitudes de los tutores, algunos casos de enseñanza y la cantidad de estadísticas que debe tener un curso básico de diseño experimental

• un glosario de términos relevantes

• un ejemplo de cómo funcionó el formato de curso de dos días en dos ocasiones de prueba, con resultados de la evaluación informal de los participantes así como sus comentarios, tanto inmediatos como un año después, indicando que fue eficaz

• un programa para un posible taller de dos días de duración, al estilo de la formación de formadores, sus elementos clave y los resultados de probarlo con una serie de tutores de ED.

## Introduction

Recent criticism of animal studies has challenged their reliability to predict effects on humans, and there is evidence that promising preclinical findings in animals that fail to translate to humans often also fail to be replicated in animal models.^1^ Poorly conducted studies yielding unreliable data have been recognised as a primary cause of the problem.^2,3^ However, years after many problems in published animal research studies were pointed out,^4^ it appears that researchers are still not demonstrating a good understanding of the key issues in experimental design (ED) such as avoiding biases (e.g. through appropriate randomisation and blinding),^5,6^ correctly identifying the experimental unit,^7^ employing adequate group sizes, and confidently using designs that are more complex than simple group comparisons.^8^ The teaching of ED should help address the issues. With adequate tuition and mentoring, researchers undertaking projects ought to be able to plan and run experiments according to sound principles, to choose an optimal design, and to analyse and interpret the results correctly. Good education in ED can greatly improve scientists’ research^9^ and give novel insights to even experienced researchers.^10^

In European Union (EU) countries ‘adequate education and training’ for scientists who design procedures and projects is required under Directive EU2010/63, Article 23.^11^ The European Commission has compiled an inventory in *Three Rs Education and Training Courses and Resources*^12^ (including many that cover the design of animal experiments) and produced a guidance document for laboratory animal science (LAS) education and training^13^ that gives a framework listing the learning outcomes (LOs) for those designing procedures and projects (Modules 10 and 11), with Module 11 expecting ‘increased proficiency’. However, the amount of time devoted to ED in courses is unclear, and the EC framework leaves much scope for variation in content, in how learners reach the LOs and in the level of competence expected. A survey of course organisers and tutors involved in courses on animal-based research carried out by FELASA (Federation of European Laboratory Animal Science Associations) and presented at the 2019 Congress,^14^ reported an average time of only 6.6 h (range: 0–80 h) for covering the design of animal experiments.

A session in the FELASA 2019 Congress discussed ways to enhance awareness of good design.^15^ This led to the setting up of a FELASA Experimental Design Working Group (EDWG) in October 2020, composed of six persons experienced in teaching ED and with a range of knowledge and experience in ED and statistical analysis (see https://felasa.eu/working-groups/id/44). The group was tasked with considering the current provision and suggesting how ED teaching might be improved and harmonised across the LAS community (see Appendix 1, Supplemental Material). The outputs given here supplement the EC framework but are not just for an EU audience. They offer anyone teaching ED a common set of detailed and assessable LOs, guidance for providing effective teaching of ED to early career researchers, and a potential programme for teachers to tutor other ED teachers who wish to improve their teaching.

## Methods

### Fact finding

EDWG Task 1 was to review ED course teaching against Directive 2010/63/EU requirements (see Appendix 1). We reviewed details in the EC inventory^12^ and the FELASA survey presentation,^14^ and the information on course content that these provided, before looking in more detail at the topics covered in the varied courses of which we had first-hand experience from running or contributing to them. These were the European Brain Research Area, BIH (Berlin Institute of Health) Quest, and Enhancing Quality In Preclinical Data Schools, I3S Porto Experimental Design courses, and FRAME (Fund for the Replacement of Animals in Medical Experiments) and FRAME-VetBionet Training Schools,^10^ all of which are open to participants across Europe, plus UK-specific courses for project licence applicants and the University of Oxford’s internal training.

Based on the topics in common in the courses and personal experience we defined the minimal knowledge and skills researchers should be able to show after a basic ED course (EDWG Task 2), expressed as LOs, and estimated that 12 h of tuition would be needed, ideally in a 16-h workshop occupying two consecutive days each with 6 h teaching and 2 h for breaks. We developed a template 2-day programme that would be flexible for different circumstances, and identified key LOs to cover if severe time constraints are imposed. We presented key material in varied formats, in two sessions at the FELASA 2022 Congress, receiving generally positive reactions and several helpful suggestions from the participants.^16^

### Testing

To confirm that the programme could be delivered and the LOs achieved in 2 days, we organised a pilot ED workshop aimed at early career researchers. We included some assessment and obtained detailed feedback. All the group took part, providing a variety of material and of presentation styles. Of the 21 attending, 12 were PhD students with wide EU representation. Feedback was sought by questionnaire after each day of the workshop, and the workshop programme and LOs refined. A second, refined, researcher workshop was later run with participants who generally had more research experience and only two of us taking part.

### Guidance

Using the experience of the pilot and our own, we drafted guidance on the content and presentation of courses (EDWG Task 3), and made recommendations on what background and skills ED tutors should ideally possess (EDWG Task 4). The guidance and recommendations form a section of this report.

### Training-the-trainers

Having established that we had an effective early career researcher tuition framework, we then trialled a 16-h, 2-day workshop in a ‘training-the-trainers’ format, which could help equip ED teachers to run researcher tuition (EDWG Task 5). Of the 20 participants, from several EU countries, some were already running ED courses (13), or had some ED teaching experience and were preparing to run ED courses (6), and there was also one statistician. The programme included reviewing of the LOs proposed and the early career researcher workshop arrangement suggested, and discussion on different formats such as group discussion sessions and quiz-type assessments. The tutors also shared their approaches to ED teaching and commented on our draft guidance and recommendations for ED courses. Their suggestions are reflected in the outputs presented here.

### One-year-on questionnaires

Follow-up questionnaires were sent a year later to attendees at the first researcher workshop, asking for retrospective judgement of the event and its effectiveness, and to those at the workshop for ED teachers, asking how their teaching may have changed since the workshop.

## EDWG outputs

### Recommended learning outcomes and a tested experimental design tuition programme

The review of ED courses listed above showed the topics commonly covered were:

- the basics of ED,
- independent replication, experimental units, pseudoreplication,
- avoiding bias: randomisation and blinding,
- reproducibility concerns,
- types of design and their pros and cons,
- hypothesis testing and the risk of false positives and false negatives,
- the meaning of significance levels and power,
- estimating numbers: power analysis, and
- reporting guidelines.

The 3Rs (replace, reduce and refine), harm–benefit analysis and welfare issues were dealt with in some courses, while others had considerable content on statistical testing and analysis of results; only a few of the courses covered the pre-registration of studies. We considered this range should be included in the basic ED course and, on the basis of our experience and the typical 2- to 3-day length of residential ED courses, at least 12 h tuition would be needed. In a 2-day face-to-face workshop, this would equate to 6 h of tuition and 2 h for breaks each day. Table 1 gives a programme in that format, but the programme could also be spread over four half-days, or as online presentations with breakout rooms for group discussion. Note that the content is progressive and that attendance at the first sessions is necessary as these deal with important issues of study quality.

**Table 1.** Recommended LOs and suggested topics for a two-day course.

Title	Learning outcomesThe learner should be able to …	Suggested topics for the session
Day 1		
Introductions40 min		Brief description of participants’ research.
Quiz 1 30 min		For example, 10 to 12 questions, with one or two from each main session, in multiple choice format with a ‘not sure’ option, designed to stimulate thought and to pick up the level of understanding at the start of the course.
General ideas 15 min	**A1** Give two examples of poor practice seen in animal experiments.	Poor reproducibility/replicability of published studies Brief overview of questionable practices Experimental design in the context of the 3Rs Types of experiment.
	**A2** Define replacement, reduction and refinement in the context of animal-based research.	
	**A3** List the key features of a good experiment.	
	**A4** Describe briefly the characteristics and appropriate use of the following types of experiments: hypothesis testing (confirmatory) exploratory pilot data-gathering or observational	
Basic experimental design55 min	**A5 State the requirements of a good objective (statement of purpose) for a single experiment and give an example**.	Stating clear objectives (experimental questions) Comparisons and controls Independent replication, experimental units, pseudoreplication Avoiding bias by proper randomisation and blinding.
	**A6 Define bias and list ways in which animal-based research is susceptible to bias.**	
	**A7 Define the terms randomisation and blinding as applied to research studies and outline how randomisation and blinding should be carried out.**	
	**A8 Define independent replication in the context of a single experiment.**	
	**A9 Define the experimental unit and give an example of one in a simple experimental scenario.**	
	**A10 Define pseudoreplication and give an example of it**.	
Break 20 min		
Group session 160 min	**A11 Identify the experimental unit in different basic experimental arrangements.**	Identification of need to randomise or blind Identification of the experimental unit in different scenarios.
	**A12 Recognise pseudoreplication in a simple erroneous experimental arrangement**.	
Animal-related issues40 min	**A13** List the important considerations in preparations for an animal study, such as those given as headings in the PREPARE guidelines**A14** List the animal welfare factors that might affect data quality in an animal study by, for example, loss of data points or increased variability.	The PREPARE guidelines The concepts of harm–benefit analysis and the 3Rs and how these influence experimental design Ways of minimising numbers Welfare and severity considerations for data quality and design Controlling variability and maximising effect.
	**A15** Define scientific endpoint and humane endpoint.	
	**A16** Give an example of implementing a humane endpoint through frequent monitoring and communication with animal facility staff.	
	**A17** Give an example of when a more invasive procedure could be acceptable because fewer animals are needed and/or better data is obtained.	
	**A18** Explain briefly the concept of the signal/noise ratio in an animal experiment.	
	**A19** List the main sources of variability in animals.	
	**A20** Give reasons for using both sexes and using animals of limited genetic variability (such as isogenic strains of rodents).	
	**A21** Explain briefly how the following may affect an experiment: stress and distress environmental conditions human factors microbiological variation housing and husbandry and give an example of how to mitigate the effect of each.	
Lunch 60 min		
Basic statistics, hypothesis testing and power60 min	**B1** Explain the difference between descriptive and inference statistics.	Basic descriptive statistics Hypothesis testing Risk of false positives and false negatives The meaning of significance levels and power Estimating numbers: power analysis.
	**B2** Explain the logic behind a hypothesis test.	
	**B3** Describe null-hypothesis testing and how it leads to risks of false positives (type 1 error) and false negatives (type 2 error).	
	**B4** Define the terms confidence interval, statistical significance, *p*-value, and statistical power.	
	**B5 Explain the connection between hypothesis testing and sample size calculation.**	
	**B6 Explain how control of experimental variability can help reduce the number of experimental units needed.**	
	**B7 Describe the use of power analysis to estimate the power of a proposed study.**	
Group session 2a: randomisation15 min	**B8** Randomise experimental units to treatments in a completely randomised design	Using MS Excel to randomise for two or more groups.
Break 20 min		
Group session 2b: power analysis30 min	**B9** Estimate the number of experimental units needed for a proposed experiment using power analysis.	Using power calculation software (e.g. G-Power) to estimate numbers and power for a two-group comparison.
Animal models and harm–benefit analysis20 min	**A22** List the criteria used to assess the suitability of a potential non-animal or animal model for biomedical investigations.	Animal models: fidelity and discrimination Considerations for a harm–benefit analysis.
	**A23** Define fidelity and discrimination and give an example of a high-fidelity model and a high discrimination model.	
	**A24** Describe briefly how a harm–benefit analysis would be carried out to meet legal requirements and ethical considerations.	
Reproducibility issues from the conduct of an experiment30 min	**C1** Define the term reproducibility in the context of animal experimentation.	Lack of randomisation and blinding in studies Internal and external validity.
	**C2** Define the term replication as applied to an experiment.	
	**C3** Define the terms generalisability and translational as applied to animal experiments.	
	**C4** List the ways in which the conduct of a study is liable to lead to poor reproducibility.	
	**C5** Define the terms internal validity and external validity.	
	**C6** Explain with examples the concepts of face and construct validity of a model.	
Close Day 1		
Day 2		
Group session 3: errors in design 30 min	**C7** Pick up design mistakes in a range of experiments.	Identifying various design errors.
	**C8** Identify opportunities for concealed treatment allocation and blinded outcome assessment in different experimental examples.	
Standard designs: picking a suitable design50 min	**D1** List different experimental designs suitable for hypothesis-testing experiments.	The concept of blocking Types of design○ fully randomised design,○ randomised block design,○ factorial arrangements,○ cross-over designs. Choosing a design: advantages and disadvantages of different designs Using NC3Rs Experimental Design Assistant.
	**D2 Describe the concept of blocking and list the advantages and disadvantages of blocking.**	
	**D3 Describe different blocking arrangements.**	
	**D4 State the main feature(s) of and give an example of each of the following designs or experimental arrangements:** **fully (completely) randomised** **randomised block** **factorial** **cross-over** split-plot	
	**D5** List the main advantages and disadvantages or potential problems with each of these.	
Group session 4: designs40 min	**D7 Identify blocks in different scenarios.**	Different scenarios for each of which a different type of design would be the most appropriate.
	**D8 Select a suitable blocking arrangement in a proposed experiment.**	
	**D9** Match the appropriate requirements to different types of experiments.	
	**D10** Identify the design described in different scenarios.	
	**D11** Select a suitable design in a proposed experiment.	
Break 20 min		
Statistical testing: analysis of variance 60 min	**B10** List the statistical tests commonly used in analysing preclinical research and describe briefly the use of each and the output obtained.	Randomisation for more complex designs Power analysis for more complex designs The concept of degrees of freedom Use of the resource equation How variability is analysed: ANOVA Dealing with different types of data and non-normality Non-parametric tests Statistical tests suitable for different designs Common post-tests and their usage.
	**B11** Explain analysis of variance (ANOVA) and the layout of an ANOVA table.	
Group session 5: testing40 min	**B12** Select the right test for a particular experiment.	Identifying which analysis and test are suitable for various designs and data Using suitable software to do a *t*-test, 1-way and 2-way ANOVA Using post-testing.
	**B13** Correctly input blocks into a statistical analysis.	
	**B14** Correctly input data from different designs into a statistical analysis.	
	**B15** Estimate the number of experimental units for a proposed experiment using the resource equation.	
Lunch 60 min		
Reproducibility issues 2:30 min (with pre-video)	**C9** Describe the process of registration of an animal-based study and give the advantages of pre-registration.	Pre-registration of studies Reproducibility networks What is meant by p-hacking and HARKing Good reporting; ARRIVE guidelines Publication bias and lack of negative results.
	**C10** Name one reproducibility network and indicate how a researcher can contribute to it.	
	**C11** Define the terms p-hacking and HARKing and give reasons why these are considered unacceptable practices.	
	**C12** Give examples of misleading presentation of data.	
	**C13** List the advantages of full disclosure of data.	
	**C14** Define raw data, processed data and meta-data.	
	**C15** Give reasons why safe storage of data and traceability are considered important.	
	**C16** List the components of good reporting as illustrated by the ARRIVE guidelines.	
Quiz 2:40 min		Same format as in Quiz 1, with perhaps a mix of the same questions and new ones on some topics at a similar level of difficulty.After submission of responses, discussion of possible answers and of which are thought best.
Group session with tutors 1: 30 min		Discussion of participants’ research.
Break 20 min		
Group session with tutors 2: 50 min		Discussion of participants’ research.Feedback on the workshop.
Quiz 2 results and closing remarks 10 min		Presenting the spread of marks in the group for both quizzes, and then anonymous individual changes in score. Brief discussion of any questions commonly mis-answered.
Close Day 2		

Here, the recommended LOs are placed within the framework of sessions used by the EDWG to test they could be taught in a 2-day course. The first column lists session titles or formats, presented in a suggested sequential order to reflect the progressive aspect of the course, with estimates of session length (in minutes). The LOs that learners should be able to achieve after each session are given in the second column, and the key LOs for a course covering the minimal topics (see Guidance) are in bold. For information, and to help EU course organisers, the European Commission’s Education and Training Framework Learning Outcomes^13^ that most closely match the LOs specified for each session are indicated in the Supplemental Material. The last column suggests topics or formats appropriate for the session.

Most courses reviewed included some sessions with group discussions or practical exercises. We have found that these are an important element of an ED course that encourages active learning and we have included several in the suggested programme.

To aid clarity and harmonisation, the elements within a topic are listed in Table 1 as detailed LOs, and these are grouped in sessions with suitable titles and timings for the programme. Appendix 2 (Supplemental Material) lists some currently available relevant teaching material that could be used for each session.

Testing the practicality of this programme in two 2-day workshops principally for early career researchers confirmed that the LOs could be addressed effectively for that audience in the time. However, tutors should adjust the teaching to their circumstances (see the section covering course length) and not take this as a recommended universal programme.

**Figure 1.**
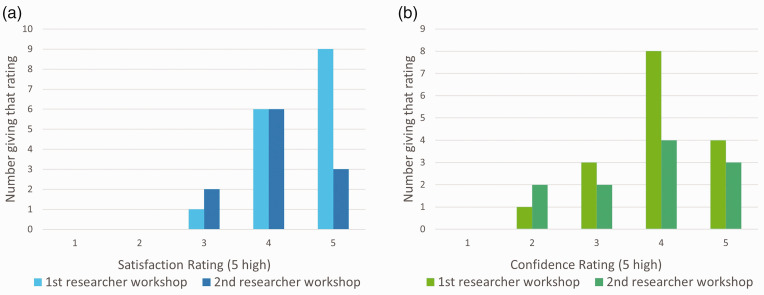
(a) Numbers rating satisfaction with the teaching from 1 (unsatisfied) to 5 (totally satisfied), and (b) numbers giving a rating from 1 (not at all) to 5 (greatly) of how much the workshop improved their confidence in designing experiments well, at the first (16 responses) and second (11 responses) early career researcher workshops.

The participants’ general ratings for the workshop are presented in Figure 1 and show a high level of satisfaction with the tuition and a good level of confidence in their ability to design. Participant feedback is fully detailed in Appendix 3 (Supplemental Material). The 12 responses from first workshop participants to the one-year-on questionnaires gave an average satisfaction rating for the course in retrospect of 4.75/5 and a mean usefulness rating of 4.33/5.

**Figure 2.**
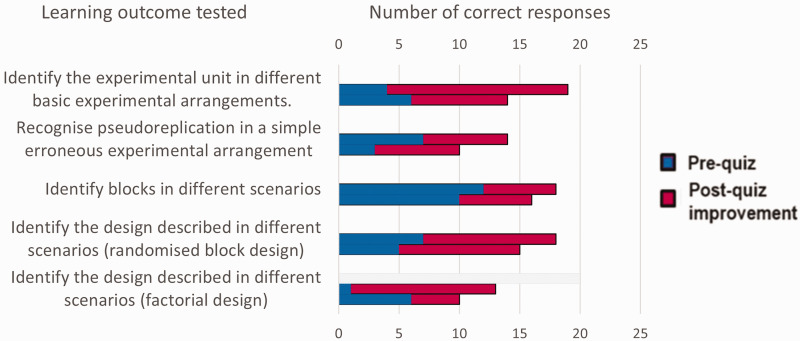
Numbers of correct responses to questions testing particular learning outcomes in anonymous pre- and post-quizzes, showing post-quiz improvements. Top bars are from the first researcher workshop (19 responses) and lower bars are from the second (16 responses).

The pre- and post-quizzes, in which the responses were anonymous, were primarily for giving learners confidence that they had advanced in their understanding and to provide further learning from discussions of the answers after the post-quiz. However, they also gave an indication that several of the LOs were achieved by 60% of the participants, as can be seen from Figure 2. The increased facility with which participants used the terms and discussed the concepts towards the end of the workshops provided further indications that a formal assessment would most likely have shown that several of the LOs were achieved.

The times suggested for each session in Table 1 reflect how long the session actually took in the first researcher workshop. A break, ideally followed by a further tuition session, was found to be necessary after the post-quiz to allow for the quiz responses to be marked and put in an appropriate form for presentation at the end of the workshop.

### Trialling a programme for experimental design tutors

We considered the key elements of a programme designed for tutors should include

- Topics to cover and LOs expected. Anyone providing the suggested tuition would need to have confidence in teaching to the LOs in Table 1, so the workshop should include sessions for participants to discuss and comment on the specified LOs.
- Exposure to a range of material and teaching approaches, in particular to the use of scenarios for group discussions and informal assessment, and discussion among the participants of their own ideas for teaching ED. The researcher workshop programme shown in Table 1 could be put forward as one that has been tested and found effective that tutors could use for their own courses.
- Practice in constructing simplified realistic research scenarios of the type used in the pilot researcher workshop, which were found to be liked and effective.
- Basic statistics and power analysis, with discussions of how these topics can be conveyed in a way that is understandable and clearly relevant to the local researcher audience.
- Assessment, both informal, as in the pre- and post-quizzes in the researcher workshop programme or audience polls during talks, and any formal assessment needed.

The workshop for those teaching or expecting to teach ED followed a programme including all these elements (see Appendix 4, Supplemental Material). A session on ‘flipping’ introduced a teaching approach (‘flipped classroom’) that aims to facilitate deep learning, in which learners are required to study informative material prior to the tutored time, to allow the tutored time to be dedicated to active learning, such as problem-solving, case studies and group discussions. Participants also shared experiences of running a week-long course and using the Experimental Design Assistant. On the basis of comments from the participants during the workshop and afterwards, it was judged a useful forum for discussion of the LOs and the effectiveness of various teaching material and different approaches.

The tutors considered teaching to the recommended LOs in the session times indicated in Table 1 to be challenging. The LOs were accepted as a suitable list for a basic ED course, although some of the tutors reported that they might phrase them differently. Some would omit LOs relating to harm–benefit analysis, refinement and welfare, while others felt their inclusion was really valuable. We present the full range of LOs, and discuss in sections of the Guidance variations such as covering animal-related LOs A13 to A17 in other parts of a course covering all 3Rs. Key LOs that we thought should be in a very basic ED course (see Guidance) are identified in bold in Table 1.

It was clear from the feedback from early career researchers (see Appendix 3, Supplemental Material) that group discussion was much appreciated as a learning experience. The tutors were able to suggest some realistic experimental scenarios that could be used for group discussion or in the pre- and post-quizzes (see Appendix 5, Supplemental Material).

**Figure 3.**
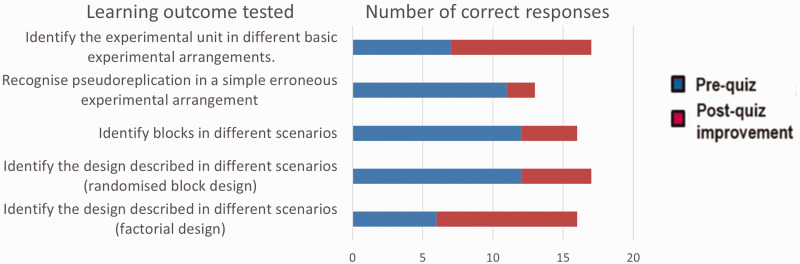
Numbers of correct responses to questions testing particular learning outcomes in anonymous pre- and post-quizzes given by participants at the ED tutor workshop (17 responses).

After participating in the anonymous pre- and post-quizzes themselves, several tutors mentioned seeing value in this approach and some have subsequently used it in their own courses. The quizzes they were given included questions testing the same LOs as shown in Figure 2, and their responses are shown in Figure 3.

Figure 3 shows that although the tutors did better than the early career researchers in the pre-quiz, they still improved notably in the post-quiz, after seeing some of the material the EDWG had prepared for use in researcher tuition.

The tutors also provided some useful reflection on statistics sessions. These are included in the responses to open questions circulated during and after the event. A summary of these is given in Appendix 6 (Supplemental Material).

The nine responses to the questionnaire sent to all attendees a year after the workshop indicated that eight had made some changes to their teaching as a result of the workshop, with five mentioning altered content, four the use of scenarios, three the introduction of pre- and post-quizzes and two the use of videos. The responses could have been anonymous, however, the four who chose to identify themselves were from different countries.

These one-year-on responses reflected the discussions during the workshop in which participants focussed more on the usefulness of the LOs and teaching approaches to their own teaching than on training other tutors to run researcher tuition with a programme like that in Table 1. There was also quite a difference between tutors in their fluency in the subject, which is seen in the results shown in Figure 3. So, although developed as a 2-day workshop in the training-the-trainers format, this trial event brought home to us that a single 2-day workshop is insufficient to bring teachers with different experiences of ED teaching to a point where they could stimulate a rolling sequence of training.

### Guidance and recommendations for experimental design courses

#### Course tutors

- Courses are likely to benefit from having more than one tutor, particularly for topics needing different knowledge/experience. Different presenters allow changes of rhythm and educational approaches, with opportunities for debate between them and general discussion, and can be helpful for answering questions from groups in the discussion sessions. However, it is important that different contributions are well co-ordinated and that each tutor is aware of the presentation and viewpoint of the other(s).
- Tutors should be able to interact well with learners of different backgrounds, and be experienced in encouraging active learning as well as lecturing. Ideally, the tutors should between them have some background in LAS, understand about the handling and care of animals of different species, have conducted some animal experiments themselves, and have experience of an ED course run by expert tutors.
- Tutors should be confident in conveying the importance of randomisation and blinding, and of the proper identification of the experimental unit in different circumstances. They should also be able to give supervised practice in performing these, and to illustrate how fully random, randomised block and factorial experiments should be conducted. At least one of them should be confident in basic statistics and the analysis of simple designs, and able to indicate clearly when expert statistical advice should be sought.

#### Course length

- The period dedicated to the course should be appropriate for the audience. For early career researchers and mixed-experience participants, at least 2 days or the equivalent should be allowed (16 h including 2 h/day for lunch and refreshment breaks) to cover the material needed for them to achieve the LOs specified. Provided they are progressive, with attendance mandatory at the first sessions, which deal with important issues of study quality, other formats might be four half-days if, for example, embedded in a 3Rs course, in which some animal-related LOs were covered in other sections, or a very basic 4- to 6-h tuition supplemented by 6 to 8 h at a later stage. Full days, however, provide more focus than shorter periods and avoid the recap time of spaced shorter sessions.
- To cover all the LOs in Table 1, although the EDWG found 2 days can be sufficient, much depends on the experience of the tutors and of the participants. When asked whether the course should last 3 days, after the first workshop 9/16 participants answered ‘yes’ and 3 ‘no’; after the second, 3/11 of the generally more experienced participants said ‘yes’ and 4 ‘no’ (see feedback responses in Appendix 3, Supplemental Material).

#### Course programme

- This should take a sequential approach, progressing from the basics of good study quality for a completely randomised two-group comparison to the more complex designs and their usage, with examples.
- The sessions should include group work. Providing groups with realistic experimental scenarios that they can analyse and discuss was found to be much appreciated by participants and has proved a very effective teaching tool. Therefore, sessions allowing for a good discussion of scenarios should be part of the mix of approaches. Group members learn from each other and discussion brings out misunderstandings. Mixed-background groups, with researchers varying in experience and/or the types of research and/or species used, are good for this, and randomisation of participants to groups can provide an adequate mix, although some adjustment to particular circumstances may be needed. On the other hand, making up groups with people of similar background can give more in-depth consideration. The plenary sessions following group work need to be focussed, allowing for some uncritical discussion but avoiding over-lengthy presentation from any group.
- Ideally the tuition should be face-to-face, but can be done effectively online with breakout groups for the discussion sessions. Face-to-face courses are better for interaction with tutors and between participants.
- The course could comprise a series of cycles of presentation, group discussion, plenary. In each, a presentation (or prior reading or individual exercises), directed at enabling achievement of a set of LOs, would be followed by group discussion, which reinforces the material, and end with a plenary session that brings together the various strands in the LOs.
- Another approach, particularly for groups with similar backgrounds, could be to ask one or two group members to outline their own projects and for the group to discuss suitable designs.

#### Course content

- The LOs that can be expected from 12 h of tuition are given in Table 1 within the context of the programme the authors used for a 2-day workshop primarily for early career researchers.
- Key (basic level) LOs are shown in bold in Table 1, and if, for example, only a short course is feasible, ED teaching should aim to provide learners with an

 ○ understanding of the importance of randomisation and blinding,
 ○ ability to identify the experimental unit in different experimental circumstances,
 ○ understanding of the concept of blocking, with examples of different blocked designs,
 ○ ability to perform power analysis for a simple proposed design.

- Organisers should consider extending beyond the suggested 12 h ED tuition to give further time on the PREPARE guidelines or on reporting, as in the ARRIVE guidelines, for which practical exercises or group work sessions may be suitable. In addition, a longer course might usefully have further sessions on statistics, including analysis of covariance and mixed-effects models.
- The terminology used should be clear and consistent and any ambiguities of terms (e.g. endpoint, effect size, random factor/effects, reproducibility/replicability) explained. A glossary available to refer to throughout the course would be helpful. A suitable glossary is provided here as supplemental material.
- Statistics sessions should be included but as a minor part of the course (for example 3 to 4 h in a 2-day workshop).

 ○ These should include sufficient reminders of, or introduction to, the idea of hypothesis testing to provide understanding of:

   1. the basic concepts involved in assessing the confidence to be placed in experimental results;
    2. the meaning of *p*-values; and
     3.  how to determine the numbers of experimental units needed.

 ○ The workshop should also include discussion of the principles behind, and the assumptions involved in, two-group comparisons and analysis of variance testing, with an indication of appropriate tests for different designs and data.
 ○ The sessions should also enable participants to communicate effectively with a statistician.

- Examples should include several species *–* fish and large animals as well as rodents. While many examples can be tailored to the particular audience, inclusion of examples from various types of work gives a wider appreciation of the value of good design.

#### Assessment

- Pre- and post-quizzes have proved very useful for measuring improvement and for self-evaluation, but also make good educational tools. It is important to allow sufficient time to explain and discuss the answers expected.
- Anonymous quizzes can also be useful as mid-course teaching tools.
- Formal assessment at the end of the course may be needed for certification and helps focus people’s minds during the workshop; however, the questions must clearly arise from the material covered in the sessions. The questions in the formal assessment should be similar in format to those in any quizzes used, so that the quizzes act as practice for the formal assessment, and discussions of the quiz answers help raise confidence in doing well in the formal assessment.
- Course evaluation. To alert to matters to follow up on and to improve future delivery, organisers should seek anonymous feedback after the course, including eliciting what topics participants wish had been covered. In addition, the usefulness of the course could be evaluated after 1 year.

#### Practical matters

- Administration and logistics are important. Our experience is that it is not uncommon for practical aspects to be neglected, with the timing of sessions affected and attendees being dissatisfied with their ability to see slides or hear speakers. The room booked should allow for group discussion (difficult with fixed seating, as in a lecture theatre) and preferably permit quick movement from watching a presentation to discussing in groups. Room size and acoustics should be sufficient to avoid cross-talk between groups, and lighting and screen size should allow projected material to be viewed from all the group areas.
- Refreshment breaks should be long enough to allow participants to talk informally to tutors and have an adequate comfort break (taking into account local facilities). Putting refreshments in or adjacent to the room and having lunch available nearby encourages interaction between participants and helps good time-keeping.
- For residential courses, joining instructions giving clear directions to the venue and information on local transport help ensure prompt starting, an evening networking event has been found popular, and accommodating gathered participants in the same hotel is helpful.

## Discussion

The set of LOs produced has been phrased to make them readily assessable through a variety of formats. They are intended to be clear about what is expected so that some consistency of assessment can be achieved among tutors/assessors from diverse backgrounds. With reference to the LOs specified in the European Commission education and training framework^13^ for the training of personnel designing procedures and projects (Function B staff), the LOs in Table 1 cover – and expand on – all Module EU-10 LOs 1 to 7, much of Module EU-11 LOs 3 to 9, plus LOs 11.10 and 11.15–11.17, but do not cover legal issues (LOs 11.1 and 11.2), or rehoming and local arrangements (11.18 and 11.19), though we reference the PREPARE guidelines for more information on logistics, among other things. LOs 10.8 and part of 11.4 call for researchers to recognise their limitations and when to seek expert advice, and it was expected that this would be made clear in sessions in the suggested course programme. The one-year-on researcher feedback indicated that many felt the workshop had made them more aware of when to contact a statistician for specialist advice on experimental problems.

The feedback responses from both researcher workshops provide confidence that the LOs and the content of the 2-day programme were appropriate for the target audience, and that the suggested programme could be a suitable template for ED teaching in various formats. However, it is progressive, with later sessions presuming attendance at the earlier ones, so those participating need to commit to attending from the beginning.

The length of the course is an important consideration. In the first researcher workshop the content was covered in 2 days, but that was with good local organisation, tutors experienced in ED teaching who are skilled at keeping to time, and with participants who were able to keep up with the pace set. In the second researcher workshop, local circumstances made keeping to the 2-day programme difficult; yet, despite the difficulties the programme produced effective learning and a worthwhile outcome. However, 3 days or equivalent spaced half-days may be generally more realistic for teaching the full range of topics. A substantial number of the researchers considered the workshop should have lasted 3 days, and the participants at the tutor workshop also thought covering the material suggested in 2 days would be challenging for them.

The topics to be covered if participants are to achieve the LOs (see Table 1) require a wide range of knowledge and it is likely that at least two tutors would be needed for a course of this nature. Good co-ordination between different tutors can make a big difference to the smooth running and educational effectiveness of a course, and the recommendations emphasise the importance of this. This approach was used for the pilot researcher workshop, which was rated highly for satisfaction with the teaching. The sessions were presented by different EDWG members using different teaching styles, therefore, the level of satisfaction is not likely to be linked to a particular presenter or style.

Some good text and video material is available (see Appendix 2, Supplemental Material) and EDWG members have found in their own teaching that pre-study of texts or videos can be a useful way of dealing with the range of topics in less time or with limited tutors. The Education and Training Platform for Laboratory Animal Science (ETPLAS)^25^ has online courses for Modules 10 and 11 of the EC framework that may be suitable. The effectiveness of pre-course material may depend on how conscientiously it is studied, and initial testing in the course itself helps encourage that. Notably, all but one of those at the first researcher workshop had viewed the pre-workshop videos for the statistics sessions and this may be a factor in how well those sessions were appreciated. However, those at the second researcher workshop, which relied extensively on these videos, generally wanted more direct teaching. Incidentally, the degree of appreciation of the statistics sessions in the researcher feedback supports our thinking that some basic statistics sessions related to animal research should be included in a basic ED course.

An important output from our background experience and from running the researcher workshops is a set of recommendations on the ideal profiles for tutors in ED, and guidance on the running of courses. The value of including group discussion of simplified realistic research scenarios is emphasised. This has long been found an effective ED teaching method,^17^ but suitable scenarios are not easy to devise, as the tutors found in the tutor workshop. There is now guidance on preparing scenarios, and also on the use of a similar approach for statistics sessions concentrating on power analysis.^18^

Teaching in ED should advance understanding of the issues, for which tutoring is likely to be more effective than just information provision. The terms ‘participants’ and ‘tutors’ have generally been used here. However, the training-the-trainers model used for more specific training can be adapted to a rolling programme for helping those teaching ED to enhance their skills. The tutor workshop programme given in Appendix 4 could provide a ready-made programme for such a series of tutor events, some of which could be online. The two sessions particular to our event (10:00 on Day 1 and 16:00 on Day 2 in Appendix 4, Supplemental Material) could be substituted by sessions relating to local circumstances. The other sessions cover key elements to help tutors, which could be used for a rolling programme in which the participants could subsequently tutor others. However our experience with running the ED teacher workshop showed more than 2 days, or more than one event, with probably a period of mentoring from an experienced ED teacher, is likely to be needed if tutors have a range of experience with ED teaching.

Although those at the ED tutor workshop have not gone on to run events to help other tutors, it is encouraging that in the year since the tutor workshop, several participants had already managed to incorporate in their teaching some of the ideas introduced to them at that event. That early career researchers gave ratings for satisfaction with, and usefulness from, the workshops covering the recommended LOs is also most encouraging.

## Supplemental Material

sj-pdf-1-lan-10.1177_00236772241295308 - Supplemental material for Teaching experimental design: outputs from the FELASA Working Group Supplemental material, sj-pdf-1-lan-10.1177_00236772241295308 for Teaching experimental design: outputs from the FELASA Working Group by Derek Fry, Manuel Berdoy, Monica Forni, Carlos Oscar S. Sorzano, Thomas Steckler and Nuno H. Franco in Laboratory Animals

## Data Availability

The only data presented in the report itself is on the attendees’ overall results in the pre- and post-course quizzes and summary responses to the anonymous questionnaires. The supplementary material gives a glossary and appendices that provide further information on the outputs reported, but without individual data. Sharing more than the overall scores for the quizzes or individual questionnaire responses would violate personal information. Any enquiries in this regard should be directed to the corresponding author.
