# Supplementary material for: Teaching experimental design: outputs from the FELASA Working Group
Source: Lab Anim. 2025 Jun 9;59(5):614–29. doi: 10.1177/00236772241295308 (PMC12583620; doi:10.1177/00236772241295308)
Supplement: sj-pdf-1-lan-10.1177_00236772241295308 - Supplemental material for Teaching experimental design: outputs from the FELASA Working Group [file sj-pdf-1-lan-10.1177_00236772241295308.pdf]

Glossary and appendices - Supplemental material for 'Teaching Experimental Design: Outputs from the FELASA Working Group'

Supplemental material: Glossary and appendices, for 'Teaching Experimental Design: Outputs from the FELASA Working Group' by Derek Fry, Manuel Berdoy, Monica Forni, Carlos Oscar Sorzano, Thomas Steckler and Nuno Franco in Laboratory Animals.

Glossary

**Term and alternatives**	**Definition**
Bias	The over- or underestimation of the true effect of an intervention. Bias is caused by inadequacies in the design, conduct, or analysis of an experiment, resulting in the introduction of error. Points where bias may occur include in selection or allocation, in handling or placing within an environment, in measurement or detection, or in reporting.
Biological variability	The part of variation in a sample which results from intrinsic biological (genetic, epigenetic, physiological, behavioural) differences between individuals, as well as in how they respond to extrinsic factors, such as the treatment, the environment, or from interaction with other replicates.
Blinding<br>Masking,	The condition of deliberately concealing the assigned treatment from the person selecting, allocating, measuring or analysing experimental material.
Block	A grouping of experimental units according to a biological characteristic (e.g. litter), environmental condition (e.g. rack shelf level) or experimental arrangement (e.g. laboratory), which is taken into account in the experimental design and analysis. While it can affect the experimental outcome, it is of no interest for answering the research question (for example, in assessing the effect of a drug there is no interest in seeing whether it has an effect on a given litter).
Completely randomized design<br>Fully randomized design<br>Single-factor design<br>Single-factor fully randomized design	The fully or completely randomized design (CRD) is the simplest type of design, in which two or more treatments are assigned completely at random so that each experimental unit has the same chance of receiving any one treatment. Only one factor is studied but there may be any number of levels of that factor being compared. For data that meets the assumptions, often analysed by a t-test (for two groups) or one-way ANOVA (for two or more).
Confidence level	The probability of not rejecting the null hypothesis when it is true. The confidence level is one minus alpha, the significance level.<br>Upper and lower confidence levels (often set at 90%, 95% or 99%) may be used to give a confidence interval or range of values in which a parameter of interest would be expected to fall.
Confirmatory experiment/study	An experiment designed to test an hypothesis conducted with scientific rigour using a formal design. It must be adequately sized, and statistically analysable. (See also exploratory experiment below)
Confounder	A source of variation that is not properly considered in the experiment's design and analysis. It is distributed non-randomly with respect to the treatment or outcome measure and so can mask, reduce or exaggerate an effect or association.
Covariate	A measure of a feature of the subject, such as body weight, which is unaffected by the treatment being applied in the experiment and which may account for some of the variability in the post-treatment results. Usually a pre-treatment measure.

Cross-over design	In this design, the same biological unit (an animal or plant) receives two or more different treatments, delivered sequentially in a randomized order. A washout period is typically required to make sure there is no carry-over effect between exposure to one treatment and the following one.<br>Such designs, when feasible (i.e. when none of the treatments has permanent effects) allow for a single biological unit (e.g. a cow or a rat) to represent two or more experimental units ("biological unit with a given treatment over a period of time"), furthering the 3Rs principle of Reduction. It moreover allows for blocking, if each time period is treated as a block, with all different treatments being assigned to independent biological units.
Descriptive and inferential statistics	Descriptive statistics are used to summarise the data. They generally include a measure of central tendency (e.g. mean or median) and a measure of spread (e.g. standard deviation or range).<br>Inferential statistics are used to make generalisations about the population from which the samples are drawn. Hypothesis tests such as ANOVA, *t* tests, or, for data that do not fit the assumptions for parametric tests, Mann-Whitney tests are examples of inferential statistics.
Effect size	Quantitative measure of difference between groups, or strength of relationships between variables.
Effect size (standardised), signal to noise ratio, Cohen's d, Cohen's f, Delta	The effect size as above, expressed in the context of the observed variability of the outcome measure, usually as the ratio between the measured difference or difference of interest (signal) and the standard deviation (noise). Large group differences may yield small standardised effect sizes if variability is also large, and conversely small differences might be 'statistically significant' if variability is low and/or the precision of the estimate is high. The measure of effect size will depend on the type of comparison and variable. For example, for a two-group comparison of a continuous variable, Cohen's *d* and Glass' delta are appropriate, among others. For the same variable, effect size for a comparison between two or more groups, can be expressed as Cohen's *f*, Eta squared, or omega square, among others. While some text books and software propose for social sciences a Cohen's d of 0.2, 0.5 and 0.8 as, respectively, small, medium, and large effect sizes, for inbred laboratory animals under controlled conditions, Cohen's d values of 0.5, 0.8 and 1.5 have been proposed as more appropriate.
Experimental unit Biological replicate	Biological entity subjected independently of all other units to an intervention and any influences throughout the experimental procedure. It should be possible to assign any two experimental units to different treatment groups and for them to be separately randomized for environmental and other influences. It is the unit of randomisation.
Exploratory experiment/study	An experiment to explore an idea, gain information, or generate initial data. It may be used to formulate hypotheses, but these must be tested subsequently in adequately powered confirmatory experiments (see above).
External validity	Extent to which the results of a given study enable application or generalisation to other studies, study conditions, or animal strains/species, or are translatable to humans.
Factor	A variable controlled by the researcher that can be used to quantify a source of variability in the experiment. Examples are treatment, sex, age, genetic status.
Factorial design - fully randomized	In factorial designs, the effect of two or more factors (for instance drug treatment AND sex AND strain) and their interactions is of interest. If a two-way (or three-way) ANOVA test is used to analyse data, rather than analysing one factor at a time, great power can be achieved with small samples, furthering the 3Rs principle of Reduction. Also, only these designs allow

	detecting both the main effects of the fixed factors being studied and possible interactions between these factors.
False discovery rate (FDR)	The expected proportion of false positives (type 1 errors), expressed as the ratio of the expected number of false positives over the total number of positives (false positives + true positives). It is often used as a measure to estimate methods of controlling for false positives (type 1 errors) due to multiple comparisons.
False negative<br>Type II error	Statistically non-significant result obtained when the alternative hypothesis is true. (So **not** finding an effect when it **is** there.)
False positive,<br>Type I error	Statistically significant difference obtained when the null hypothesis is true, (So finding an effect when it is **not** there.)
Hypothesis testing	Statistical procedure for analysing the data obtained from an experiment and deciding whether to reject or not the null hypothesis at the sight of the accumulated experimental evidence. We may: 1) reject the null hypothesis (and accept that the alternative hypothesis is true), or 2) fail to reject the null hypothesis (but in this case we cannot state that the null hypothesis is true or the alternative hypothesis is false).
Humane endpoint	The level of severity or other criterion at which an animal should be taken off an experimental procedure, for ethical reasons. In addition to the severity experienced being more than the data to be collected justifies, other criteria may be that the purpose of the experiment either has been achieved or has become clearly unachievable.
Independent variable<br>Predictor variable	Variable that the researcher either manipulates (treatment, condition, time), or is a property of the sample (sex) or a technical feature (batch, cage, sample collection) that can potentially affect the outcome measure. Independent variables can be scientifically interesting, or nuisance variables.
Inferential statistics	See Descriptive and inferential statistics above.
Internal validity	Extent to which the results of a given study can be attributed to the effects of the experimental intervention, rather than some other, unknown factor(s) (e.g. inadequacies in the design, conduct, or analysis of the study introducing bias).
Levels	The extent to which a factor is varied, at discrete steps or categories. (e.g. for the factor treatment, levels could be vehicle/agent 1/agent 2; for the factor sex it will be male/female; for genotype it could be wild-type/genetically-altered)
Measurement (analytical) variability	Variability from variation in measurement, the handling or interpretation of samples, and technical variation of equipment used.
Nuisance variable	Variabilities that are not of primary interest but should be considered in the experimental design or the analysis because they may affect the outcome measure and increase variability.
Null and alternative hypotheses	A null hypothesis describes a possible state of nature, namely that there is no effect, such as a difference between groups or an association between variables. For example, a null hypothesis might be that there is no difference between treated and untreated experimental groups.<br>The alternative hypothesis describes an alternative state of nature, namely that an effect does exist. For example, that one experimental treatment does have a measurable effect compared to another.
Observational unit<br>Technical replicate	The multiple experimental measurements that could be obtained from the same experimental unit by measuring it multiple times. For instance, several aliquots of the liver of the same animal, or two measurements of the gene expression of the same tissue of the same animal.

Appendix 1: The Working Group's Tasks

Outcome measure<br>Dependent variable<br>Response variable	Any variable recorded during a study to assess the effects of a treatment or experimental intervention.
Pilot study	A small study used to test practicalities (e.g. whether a parameter can be measured in the first place, a phenotype is observed, a test yields useful data, or if it can be done in the allocated time) and to note any adverse effects on the animals. Pilot studies can show sources of variability but use numbers too small to give a good quantitative estimate of variability, and hence should not be relied upon for sample size calculation.
Positive predictive value (PPV)	The probability that a rejection of the null hypothesis is correct based on the experimental data (i.e. probability that a detected effect is indeed true). PPV=1-FDR
Power	The probability of an experiment allowing the null hypothesis to be correctly rejected. For testing for an effect, it would be the probability that the experiment would detect an effect of a predefined size that really exists, and would depend on the sample size, the variability, and the level of confidence required.
Primary end point/outcome	The dependent variable on which the hypothesis is formulated and tested. It is used to evaluate the statistical power.
Pseudoreplication	When non-independent data points are treated as if they were independent, for example when multiple samples or measurements taken from the same animal, or animals in a cage where treatment is added to the food or water, are each treated as a separate experimental unit, and analysed as such. This leads to overestimating the sample size and to underpowered experiments, reducing the reliability of statistical inferences
Random and fixed effects	An experimental feature (batch, cage, laboratory, room, day of the week, etc.) that can potentially affect the outcome measure and which has more than one level. These features can be used as blocking factors.<br>For random effects the levels (e.g. the different rooms or cages) have been randomly sampled or are outside the experimenter's control.<br>For fixed effects (e.g. sex, treatment, time of day) the levels have been specifically chosen for study by the experimenter and are able to be repeated in subsequent experiments.<br>Controlling for random effects in the analysis can increase the statistical power of an experiment.
Randomized block design<br>Block design	A randomized block design is an experimental design where the experimental units are in groups called blocks, and the differences between blocks are not of interest for the study. The treatments are randomly allocated to the experimental units inside each block. In a completely randomized block design all treatments appear at least once in each block.<br>The study is split into "mini-experiments" (e.g. one for batch, week, etc.), each having all treatments, assigned randomly to experimental units. Each "mini-experiment" is deemed a "block", and analysis is only carried out once all blocks have been completed and the study is finished. This design may also be useful when an experiment needs to be broken down into smaller, more convenient, parts.<br>The design increases power by better control of variation (eliminating between block variation such as could be caused by shelf height in the rack, circadian rhythms, etc.). Using a randomized block design makes it less likely that observed effects are due to a confounding factor, increasing internal validity. It can increase generalizability (external validity) by sampling slightly different environments.

The working group would:

Repeated measures	The multiple experimental measurements that could be obtained from the same experimental unit by measuring it at multiple times. In repeated measures, time is a factor whose effect may vary (for instance, a high response after giving the treatment that decays over time), while in the observational unit the measurements are "indistinguishable" (for instance, measuring twice the same response variable obtaining different numerical values simply due to measurement noise).
Residual	The difference between a particular value and the mean value for that experimental group. One of the assumptions in statistical tests such as the t-test and the analysis of variance is that the residuals in each of the experimental groups are independently and identically distributed (normal/Gaussian distribution).
Sample size<br>N, n	The number of experimental units used. It may refer to the number in the whole experiment (usually given as *N*) or the number in an experimental group (usually given as *n*). It is important to make clear which of these is being used in an experimental protocol or description. Both should be calculated on the basis of the (standardized) effect size one would wish to detect, for a given significance level (α), and statistical power (1-β).
Secondary (or additional) outcome measures/end points	Other variables that may be collected during the experiment but for which the experimenter is not sure to have enough power to detect a statistical significance.
Signal<br>Group difference	The difference observed in the dependent variable (outcome measure). For power analysis it is the difference that is considered relevant from a biological point of view.
Significance level<br>Alpha	The probability of incorrectly rejecting the null hypothesis, i.e. rejecting the null hypothesis when it is true.
Variability<br>"Noise"	The differences between replicates in how they respond to treatment, which occur despite standardization of all known aspects, due to either biological idiosyncrasies of each individual (expected and useful for generalizability), measurement error (undesirable), or an unknown cause. It can be measured by the standard deviation of the residuals of the primary outcome measure. Variability within a sample is deemed the "noise" in the signal/noise ratio description for the standardized effect size.

1. Review experimental design courses intended to provide adequate education and training under the terms of the Directive.
2. Decide the minimal knowledge and skills researchers should be able to show after participating in a course.
3. Produce guidance on content and presentation of courses.
4. Define the desirable profile(s), in terms of background, work experience, and traits that tutors in experimental design show ideally possess.
5. Develop and trial a 16h, 2-day workshop in the "Training the trainers" pattern to help equip experimental design teachers with the skills to run 14-20h (2-3 day) courses with similar principles and practice, based on the guidance.

Appendix 2: Suitable material for the sessions of a two-day course for early career researchers

These are shown for the sessions of a two-day programme principally for younger researchers for which the LOs and topics are listed in Table 1. For some sessions, sheets using scenarios[18] or statistical exercises are suggested, with these being produced by the

session presenters. Note that the videos suggested may be updated and session presenters should look for later versions or appropriate newer ones.

**Title**	**Suitable material identified in 2023<br>(there is also much other good material online)**
**Day 1**	
Introductions *40 min*	
General ideas *15m*	Video giving general overview<br>https://www.youtube.com/watch?v=OWaDqyIdy0E<br>Video - 3Rs overview (NC3Rs 18min)<br>https://vimeo.com/289645718?embedded=true&source=vimeo_logo&owner=18760226<br>Introduction to experimental design (FRAME/University of Nottingham)<br>https://xerte.nottingham.ac.uk/play_24618#page2
Basic experimental design *55m*	Video on randomisation and blinding<br>https://mediasite.video.ufl.edu/Mediasite/Play/6094467bb2bb48ef9cd8d53d2f6cd96d1d<br>Festing et al. (2016)[19]<br>ETPLAS Online modules for EC Modules 10 and 11 [25]
Group session 1 *60m*	Scenario sheet - for examples of scenarios see Appendix 5 and Franco and Fry (2023)[18]
Animal-related issues *40m*	Video PREPARE cartoon (NORECOPA 4min)<br>https://vimeo.com/358069203<br>PREPARE Guidelines[20]<br>Website PREPARE https://norecopa.no/prepare<br>Video “Are happy animals better for science” (Science on line editor, 4min)<br>https://www.youtube.com/watch?v=GB5BSzPsLsw&t=24s<br>Lesson “The win-win-win-win scenario of a Culture of Care for animal research” (Nuno Franco, 44min)<br>https://www.youtube.com/watch?v=6AuR20SBt18<br>Lesson “Monitoring for Humane Endpoints: Developing an Appropriate Strategy” (Debra L. Hickman, 1.20h)<br>https://olaw.nih.gov/education/educational-resources/webinar-2018-09-20.htm<br>Video - Mouse enrichment and rat tickling (MAX PLANCK, 2min each)<br>https://www.mpinat.mpg.de/animal-facility<br>Webinar “Using both sexes in animal experiments” (Joint webinar from the NC3Rs and the MRC, 45 min)<br>https://www.nc3rs.org.uk/3rs-resources/mrc-nc3rs-webinar-using-both-sexes-animal-experiments
Basic statistics, hypothesis testing and power *60m*	From P Value to Power (20 mins) - Manuel Berdoy<br>https://tinyurl.com/pvaluetopower<br>Statistical experiment design for animal research (Carlos Oscar S Sorzano) [21]
Group session 2a: Randomisation *15m*	Exercise sheet
Group session 2b: Power analysis *30m*	Exercise sheet using e.g. G*Power or InVivoStat

Animal models and harm-benefit analysis *20m*	Bronstad et al. (2016)[22]
Reproducibility issues from the conduct of an experiment *30m*	https://eqipd181379605.wordpress.com/module-1-systematic-review-of-animal-study-data/
CLOSE Day 1	
**Day 2**	
Group session 3 – errors in design *30m*	Scenario sheet - for examples of scenarios see Appendix 5 and Franco and Fry (2023)[18]
Standard designs Picking a suitable design *50m*	Video including section on standard designs https://www.youtube.com/watch?v=OWaDqyIdy0E<br>Festing et al. (2016)[19]<br>Experimental Design Assistant (NC3Rs)[23]<br>ETPLAS Online modules for EC Modules 10 and 11 [25]
Group session 4 – designs *40m*	Scenario sheet - for examples of scenarios see Appendix 5 and Franco and Fry (2023)[18]
Statistical testing: Analysis of variance *60m*	Statistical experiment design for animal research (Carlos Oscar S Sorzano) [21]<br>The ANOVA: What the numbers mean and why it matters. (14 minutes) – Manuel Berdoy https://tinyurl.com/ANOVAnumbers<br>How to define factors and recognize their influence on results (Carlos Sorzano) https://www.youtube.com/watch?v=c9LWrVKFf5M
Group session 5 – testing *40m*	Exercise sheet
Reproducibility issues 2 *30m (with pre-video)*	Video includes section on registration https://eqipd181379605.wordpress.com/module-1-systematic-review-of-animal-study-data/<br>Video on questionable research practices https://www.youtube.com/watch?v=itMA5NaayKw<br>Paper on questionable research practices in statistical analysis and presentation of results https://www.psychiatrist.com/jcp/assessment/harking-cherry-picking-p-hacking-fishing-expeditions-and-data-dredging-and-mining-as-questionable-research-practices/<br>ARRIVE Guidelines[24]

Appendix 3: Details of and feedback responses from researcher workshops.

Venues and attendees

First researcher workshop, July 2022, the University of Bologna, Italy: 13 participants from around Bologna, one from elsewhere in Italy, and one each from Croatia, Czech Republic, Germany, Israel, Netherlands, Romania, and Slovenia

Second researcher workshop, June 2023, University of Naples Frederico II, Italy: 17 attendees from around Naples, including 5 from a pharmaceutical company, one Latvian.

Participant feedback

The first researcher workshop (F) followed the programme given in Table 1, except that with no participants wanting further discussion of their own research, the second group session

with tutors was usefully replaced by one on questions on the course material, and by a plenary feedback discussion. The pre-course material was statistics videos. Feedback was sought after Day 1 (F1, 20 responses) and at the end of the workshop, Day 2 (F2, 16 responses). In the second researcher workshop (S), to cope with delays from projection problems and intrusive noise, the statistics sessions in the programme were largely replaced by videos for participants to study overnight, and so a question about whether there had been enough on statistics was included in the feedback which was sought after the workshop (11 responses). Responses to the same or very similar questions are grouped together.

**Question**	Day	**Yes/ Definitely**	**Partly**	**No/Not at all**	**Undecided**
Did you manage to look at the pre-course material (be honest!)	F1	16	3	1	0
Did the group sessions help you to understand the content of the earlier talks?	F1	16	4	0	0
	F2	12	3	0	1
	S	11	0	0	0
Did you find the exercises on randomisation and power analysis helpful?	F1	13	6	1	0
Did you find the exercise on statistical testing helpful?	F2	10	3	1	2
Did the videos help understanding of the statistics sessions?	F2	10	3	1	2
	S	3	5	*	*
Should there be more statistics in an experimental design course than in this workshop?	S	7	0	4	0
Do you think the sessions could have been just as useful if delivered online rather than face-to-face?	F1	3	6	11	0
	F2	1	1	14	0
	S	0	1	10	0
Did you think the pre and post quizzes were worthwhile?	F2	14	2	0	0
	S	10	1	0	0
Do you think this course should be three days?	F2	9	4**	3	0
	S	3	3**	4	1
Do you have any comments on the sequence of the sessions and the academic level of the workshop as a whole?	S			10	
Did you think the learning outcomes were clear and covered the right content for this type of course?	F2	16	0	0	0
	S	10	1	0	0

* 3 had not watched enough to comment ** perhaps

The survey sent a year later to participants in the first workshop obtained 12 responses which gave average satisfaction rating for the course in retrospect of 4.75 out of 5 and a mean usefulness rating of 4.33. All twelve respondents thought the workshop had helped them judge proposed or published experimental work and when to consult a statistician. Ten had consulted the workshop material afterwards and had been encouraged to explore experimental design or statistics further, while eleven had used the information gained to discuss ongoing experiments with colleagues.

Appendix 4: The programme used in a workshop for ED tutors

This shows the sessions and timings used for the tutor workshop run by the EDWG at i3S, University of Porto, Portugal in September 2022, with one attendee from Belgium, Denmark, Finland, Germany, Norway and Switzerland, two from Latvia and Slovenia, three from UK, and 7 from Portugal. The programme has an early explanatory background session and a penultimate group discussion on our draft recommendations. With these substituted by appropriate local items the programme, with its mix of presentations and general and small group discussions, could form a template for a two-day workshop for helping tutors develop their teaching.

Timing	Session Title
**Day 1**	
9.00	Introductions: Participants talk briefly about themselves and their involvement in experimental design teaching
10.00	The FELASA Experimental Design Working Group's Terms of Reference and work to date
10.30	BREAK
11.00	Examples of the approach and material usable for a researcher workshop<br>1. Pre-quiz<br>2. Presentation: Issues in Experimental Design that need addressing<br>3. Group discussion: Issues and experimental units<br>4. Presentation: Some experimental design variations<br>5. Group discussion: Design options
13.00	LUNCH
14.00	Comments and discussion on the approaches and material shown so far
14.40	Approaches to teaching statistics
15.30	BREAK
16.00	Group work: Preparing scenarios for group discussion or quiz questions
16.50	Round-up of Day 1
17.00	CLOSE Day 1
**Day 2**	
9.00	Discussion of the scenarios produced by the groups
9.40	Short presentations by participants of other approaches found effective
10.30	BREAK
11.00	Short presentations by participants of other approaches found effective
11.30	Discussion: "Flipping" and the use of pre-course and online material
12.10	Post-quiz using some of the scenarios
12.30	Group work: Learning outcomes for Day 1 of a 2-day course
13.00	LUNCH
14.00	Group work: Learning outcomes for Day 2 of a 2-day course
14.40	Panel discussion: Questions on training trainers and difficulties with running a course, including logistics
15.30	BREAK
16.00	Group work: Recommendations for conduct of a two-day experimental design workshop suitable for local tutors to run
16.50	Summary and closing remarks
17.00	CLOSE Day 2

Appendix 5: Scenarios

Experimental scenarios suitable for experimental design teaching suggested by the groups at the tutor workshop. The scenario texts have been modified to accord with the principles for scenario use in teaching given in Franco and Fry (2023)[18] so that there is a clear statement of purpose, text giving the proposed comparisons and a general description of the procedure involved, a statement of the outcome measure(s), and an unambiguous quiz question or one or two questions for group discussion. For quiz questions the best response is given in italics but it is important to discuss other possibilities and why that response is considered the best.

Scenario 1

A group are studying the effect of a novel drug on a plasma biomarker. They propose to give the agent or the vehicle control to group-housed mice in the drinking water for a week after taking a baseline blood sample, and repeat the blood sampling a day after the week's treatment. The levels of the biomarker in the two samples will be compared. To determine the dose of drug received the water consumed from each cage drinker will be measured daily.

Quiz question options

In this experiment is the experimental unit

A. the individual mouse,
B. *the group of mice in a cage*,
C. the biomarker level, or
D. the drug dosage?
E. I don't know

[and with extra text …]

Water consumption shows each cage group received the same dose of drug or vehicle. If the mice were housed 3 to a cage, and 6 cages received drug and 6 vehicle would the group size for statistical analysis be

A. 3,
B. 6
C. 18, or
D. 36?
E. I don't know

Questions for discussion

How would you suggest the group carry out this experiment?
How would you estimate the number of mice needed?

Useful discussion points

Variability of dosage
Randomisation of treatment allocation and cage positioning
Use of both sexes
How to do a power analysis.

Scenario 2

To determine the best sperm cryopreservation protocol for *Xenopus laevis, a*liquots of testicular material from different males which have been cryopreserved by 3 different methods will be compared for fertilisation effectiveness against a sample from the same testis kept unfrozen but refrigerated. Eggs from different females will be subdivided into dishes with roughly equal numbers, and each dish will have added to it testicular material from one of the males given one of the four treatments (3 different cryopreservations and one refrigeration). The proportion of eggs fertilised in each dish will be determined.

Previous experiments have shown that sperm from testicular material derived from different males vary considerably in ability to fertilise and that there is also much variation in maturity and ability to be fertilised between the eggs from different females.

Quiz question options

What would be the best experimental design for the study?

- *A - A randomised block design*
- B - A completely randomised design
- C - A factorial randomised design
- D - A factorial randomised block design
- E - Not sure

Is the experimental unit-

- A the female
- *B the dish*
- C the male, or
- D the cryopreservation technique?
- E not sure

Questions for discussion

How would you suggest the group carry out this experiment?
How would you estimate how many males and how many different females to use?

Useful discussion points

Various designs that might be used.
Randomisation in a complex experiment like this.
Different approaches to estimating numbers.

Scenario 3

Quiz question

A researcher wants to determine the extent to which two plant extracts prevent the development of non-alcoholic fatty liver disease. Mice will be housed 4 to a cage with males and females in separate cages and given a diet known to induce fatty liver disease. On a random basis the diet in the cage hoppers will have added the first extract, the second extract or the extract medium. After 16 weeks the animals will be euthanased and the extent of fatty liver disease in each liver determined by a standardised histological assessment on 10 sections.

Question options

Is the experimental unit-

- *A the cage group*?
- B the individual mouse?
- C the liver section?
- D not sure

What would be the best experimental design for the study?

- A - A randomized block design
- B - A completely randomized design
- *C - A factorial randomized design*
- D - A cross-over design
- E - Not sure

For group discussion

A researcher wants to determine the extent to which two plant extracts prevent the development of non-alcoholic fatty liver disease. Her proposal is that mice would be housed 4 to a cage and given a diet known to induce fatty liver disease. The diet in the cage hoppers would have added the first extract, the second extract or the extract medium. After

16 weeks the animals would be sacrificed and the extent of fatty liver disease in each liver determined by a standardised histological assessment on 10 sections.
How should she plan the experiment?
Useful discussion points
Randomisation of treatment allocation and cage positioning
Use of both sexes
How to determine the numbers needed

Scenario 4

For quiz
To determine the cytokine effect of a new drug, researchers wish to compare high and low doses of the agent against vehicle control using singly-housed beagles. Plasma cytokine levels 1 day after drug or vehicle injection will be measured. A pilot study has shown that there will be no drug left in a beagle 10 days after injection.
What would be the most efficient design (using fewest dogs) for this experiment?

A – A completely randomised design
B – A cross-over experiment
C – A factorial experiment
D – A randomised block experiment
E – I am not sure.

For group discussion
Researchers wish to determine the variation of plasma levels and cytokine response with time after injection of a new drug. They propose to compare high and low doses of the agent against vehicle control using singly-housed beagles. Blood samples will be taken to determine plasma levels every 24h after drug or vehicle injection for 6 days. A pilot study has shown that there will be no drug left in a beagle 10 days after injection.
How should they plan the experiment?
Useful discussion points
Randomisation of the beagles to the sequence of injections (and therefore sampling) for each treatment.
Use of a single person for all the injections and a single person for all the sampling, or randomising the people used.
Use of both sexes
How to determine the numbers needed

Appendix 6. Open questions circulated to those at the tutor workshop with summaries of the responses given

What is the goal of the statistical teaching? (learning to do the calculations, learning to look for help, a mixture of the two, anything else?)

1. Animal researchers should have statistical knowledge to understand limitations, assumptions, and opportunities in their research. They need statistical knowledge to avoid post-hoc hypotheses, inadequate data, poor power calculations, and other issues.
2. They should know the meaning of fundamental statistical parameters and their relationships, such as experimental unit, sample size, effect size, power, and significance value.
3. Understanding principles like randomization, blinding, and experimental units, as well as efficient designs like factorial and blocked designs, is important.
4. They should be able to translate statistical test results into understandable language and design simple studies that may require assistance from a statistician.

5. Researchers should be able to identify strengths and weaknesses in studies they review and provide advice for improvement.
6. Researchers should learn how to correctly analyse data using appropriate methods, interpret data from other papers, and know when to seek help.
7. They should be aware of the probability of false negatives and false positives, and possible factors that may affect their results.
8. Teaching should emphasize on conceptual understanding, making appropriate inferences, determining suitable statistical analysis methods, and seeking help early in the research process. It should also promote critical thinking and awareness of biases, an appropriate choice of controls and experimental units.
9. Statistical education should be a positive experience for students, providing real-life examples, and influencing decision-making after statistical analysis.

How many hours do they need to learn to do the calculations by themselves?

1. Training should have an outcome-based approach, with the number of hours varying based on participants' differences and the complexity of the technique. Typical lecturing times mentioned range from 4, 6, 12, to 20 h. However, beyond the basics, it is expected that a two-days course is not enough.
2. There should be an emphasis on group activities and practical exercises.
3. Learning statistics in a specific area and acquiring reasonable proficiency is important, similar to mastering a surgical technique. Learning statistics may involve lifelong learning and the availability of qualified teachers (for instance, small countries may lack the latter).

How do they learn the limits of what they have learned? (how to know that I cannot use what I know to design this experiment?)

1. Difficulties may arise during data entry or interpretation, indicating limitations in knowledge. After the fact: If they go to add their data to the software and they don't know what the variables are, don't know the values it spits out mean or get a result that is so far out they don't know how to interpret it they have clearly reached a limit. Lack of awareness of limits may indicate a lack of understanding.
2. Understanding the concepts behind statistical methods should provide some understanding of their limits. Participants need to be self-critical and aware of their limits. Participants should at least reach the level of conscious incompetence (https://en.wikipedia.org/wiki/Four_stages_of_competence) after completing the training. The time required for participants to reach these stages varies.
3. Participants should seek help if they are unsure about the difference they are trying to show, which analysis to use, or how to approach the experiment.
4. Awareness of pitfalls can be achieved through examples from literature and social events. Real experience and discussions with peers can enhance experiment design.
5. Allowing participants to perform calculations and identifying critical points can be helpful. Practical examples and teaching critical result analysis are effective approaches. Simple scenarios highlighting the impact of minor changes can illustrate the importance of various factors.

Statistics (and surgery) should be performed only by highly skilled people? Only professionals? Or, on the contrary, Statistics should be like our cell phones?

1. Statistics should be taught to reduce complexity and focus on situations where basic knowledge is sufficient, while complex analyses can be done by statisticians to avoid bias. Basic use of statistics is possible without in-depth understanding, but complex features may require assistance.
2. Cooperation between scientists and statisticians is crucial from the experimental design stage.
3. The availability of highly skilled statisticians is a significant concern.
4. Mandatory consultation with professionals is recommended.

5. Some understanding of statistical concepts is necessary to avoid p-hacking and inappropriate practices.

Should we design courses at different levels? Should there be an accreditation system also for Statistical design of experiments?

1. Conceptual understanding is sufficient for experimental design, but awareness of bias is important.
2. Accreditation systems are beneficial for ensuring quality, but non-accredited courses can still provide value.
3. Modules or courses focused on specific statistical tests or calculations could supplement educational programs.
4. Courses at different levels, followed by participation in supportive networks, can bring about change and improve the quality of published work. Courses targeting pre-graduate and Ph.D. students could be beneficial.
5. The implementation of an accreditation system and determining responsibility for accreditation pose challenges.
6. Advanced design courses should be available for those interested, with a network of experts for guidance.
7. Different levels of courses and accreditation are important considerations.
8. Assessing competence in experimental design, similar to procedural competence, is desirable.

Should we hide all mathematics from the most basic courses? If yes, at which point the mathematical connection is established?

1. Understanding the mathematics behind statistical concepts may not be necessary at a basic level. Introducing mathematics in a short timeframe may lead to confusion and lack of understanding. Basic courses may focus on understanding the meaning, while design-focused courses should have a mathematical connection and practical applications.
2. Mathematics should be shown at a simple level to emphasize its existence and the need for statistical understanding (results do not 'appear from nowhere'). Including mathematics can enhance understanding for some students. Some mathematical knowledge is needed to explain concepts like ANOVA. Basic information should be provided to demonstrate that there is no complete easy way in statistics.
3. Keeping things simple and graphical is preferred, but basic understanding should be provided. Visual graphics can be used to simplify and illustrate concepts.
4. Mathematics can be introduced for individuals who want to delve deeper into the subject.
5. Linking concepts to mathematics can be beneficial as the complexity of the course increases.

What is the role of self-preparation and how to maximize course impact? (should we ask, for instance, to pass a small test before being able to join a course?)

1. Basic prerequisites should be kept low to promote inclusivity and avoid discouraging participants.
2. Experimental design should be accessible at the master's student level, serving as an entry point.
3. Specific instructions should be provided for self-preparation.
4. A prerequisite test can assist in registering for courses and provide valuable feedback to tutors.
5. Recommending pre-training and self-testing can help determine the participants' level and promote engagement.
6. Self preparation is important, and a useful way of seeding ideas to researchers before a course starts. However, many researchers may not learn effectively by this method, and may instead find it much more useful to be able to interact with an educator in person when learning. There are also myriad other reasons why a researcher may not have the opportunity to read/watch the pre-course materials in advance of a course taking place.

Self-preparation materials should be available, but not using them should not unduly hinder learning at the course itself.

7. Self-preparation is of paramount importance, if we want to keep courses relatively short. A small test before being able to join the course would be an interesting option, if people can go back to the materials and re-test, in case they failed to achieve the required mark on the first try (maybe even get some helping tips!). The importance of the test would be to make sure the trainees have gone through the pre-course materials.
8. Self-preparation is crucial, and a small test before the course can be helpful for assessing understanding and guiding the teaching process. Individual readiness assurance tests ensure that all participants are at an equal minimum level, benefiting the entire course. Self-preparation increases the information participants can gather and maximize their interaction with course experts.
9. Pre-training with recorded videos and feedback forms can aid in identifying areas of difficulty and understanding. Gamification and interactive slides/tests can enhance self-preparation.

What should institutions do about the problem?

1. Institutions should actively support training researchers at both basic and higher levels, including hiring individuals with relevant background and experience. Financial support should be provided for personnel training in animal research and experiment design. Accessible modular training in statistical methods for the biosciences should be provided, preferably to an accredited standard.
2. Introduction to experimental design should be made compulsory at a certain module level.
3. Promoting good research practice and integrity should be a priority, with a focus on reproducibility networks and ethical considerations in animal research.
4. Institutions may require enforcement through legislation, recommendations, journal policies, and funding policies.
5. Collaboration between scientists and statisticians should be facilitated through meetings and clear communication of experiment goals and designs.
6. Workshops and teaching during PhD programs can be promoted, and designated individuals within institutions should be available for researchers to discuss experimental design. Institutions can establish officers (similar to the animal welfare officer) with expertise in experimental design and research integrity, working alongside statisticians.
7. Mandatory pre-registration of experiments, with review by a competent group at a European level, should be implemented to improve the use of animals in research.

What should ethical committees do about the problem?

1. Ethical committees should ensure that protocols provide enough information for assessing the planned research and the appropriateness of statistical analysis methods.
2. Ethical approval processes should ask for better experimental design information, possibly based on existing standards like the ARRIVE guidelines.
3. Ethical committees should include qualified statisticians among their members.
4. Ethical committees should verify the outcome and output of research, such as checking papers and engaging with principal investigators to improve experimental design.
5. There is a need to prevent exploratory studies from being treated as confirmatory, addressing issues of p-hacking and HARKing. Openly inquire about hypotheses and previous knowledge.
6. Ethical committees should promote training actions and emphasize the importance of statistical training at the institutional level.
7. Research ethics committees can be involved in educational activities, organizing meetings or providing recorded videos on common problems and mistakes in animal research proposals.
8. Ethical committees should ask for pre-registration of the studies.

What should FELASA do about the problem?

1. Create recommendations, collect relevant articles, short videos, and provide teaching materials and workshop training.
2. Prepare recommendations on course contents, learning outcomes, and trainer requirements.
3. Consider accrediting or recommending statistics modules in specific areas.
4. Develop a minimal content index and a final test.
5. Organize and support an expert group to review projects and train the trainers.
6. Establish a platform or network of experts in specific types of experimental designs for advice.
7. Support trainers through workshops, common databases, and sharing experiences.
8. Continue highlighting the importance of robust experimental design and comprehensive reporting.

Appendix 7

Matching of Table1 Learning Outcomes to those in the EC Module Fraamework

. **Table 1 Session**	**Table 1 Learning Outcomes** The learner should be able to…	**European Commission's Education and Training Framework Learning Outcomes that most closely match**
General ideas	**A1** Give two examples of poor practice seen in animal experiments.	**11.3**
	**A2** Define replacement, reduction and refinement in the context of animal-based research.	**11.10**
	**A3** List the key features of a good experiment.	**11.3**
	**A4** Describe briefly the characteristics and appropriate use of the following types of experiments:<br>hypothesis-testing (confirmatory)<br>exploratory<br>pilot<br>data-gathering or observational	**11.3**<br>**11.6**
Basic experimental design	**A5 State the requirements of a good objective (statement of purpose) for a single experiment and give an example**.	**11.3**
	**A6 Define bias and list ways in which animal-based research is susceptible to bias.**	**10.3**
	**A7 Define the terms randomisation and blinding as applied to research studies and outline how randomisation and blinding should be carried out.**	**10.3**
	**A8 Define independent replication in the context of a single experiment.**	**10.4**
	**A9 Define the experimental unit and give an example of one in a simple experimental scenario.**	**10.4**
	**A10 Define pseudoreplication and give an example of it**.	**10.4**
Group session 1	**A11 Identify the experimental unit in different basic experimental arrangements.**	**10.4**
	**A12 Recognise pseudoreplication in a simple erroneous experimental arrangement**.	**10.4**

Animal-related issues	**A13** List the important considerations in preparations for an animal study, such as those given as headings in the PREPARE Guidelines<br>**A14** List the animal welfare factors that might affect data quality in an animal study by, for example, loss of data points or increased variability.	**11.10 to 11.15**
	**A15** Define scientific endpoint and humane endpoint.	**11.16**
	**A16** Give an example of implementing a humane endpoint through frequent monitoring and communication with animal facility staff.	**11.16**
	**A17** Give an example of when a more invasive procedure could be acceptable because fewer animals are needed and/or better data is obtained.	**11.17**
	**A18** Explain briefly the concept of the signal/noise ratio in an animal experiment.	**10.2**
	**A19** List the main sources of variability in animals.	**10.2**
	**A20** Give reasons for using both sexes and using animals of limited genetic variability (such as isogenic strains of rodents).	**10.2, 11.3**
	**A21** Explain briefly how the following may affect an experiment,<br>stress and distress<br>environmental conditions<br>human factors<br>microbiological variation<br>housing and husbandry<br>and give an example of how to mitigate the effect of each.	**10.2,<br>11.3**
Basic statistics, hypothesis testing and power	**B1** Explain the difference between descriptive and inference statistics.	**11.3**
	**B2** Explain the logic behind a hypothesis test.	**10.5**
	**B3** Describe null-hypothesis testing and how it leads to risks of false positives (“Type 1 error”) and false negatives (“Type 2 error”).	**10.5**
	**B4** Define the terms confidence interval, statistical significance, p-value, and statistical power.	**10.5**
	**B5 Explain the connection between hypothesis testing and sample size calculation.**	**10.6**
	**B6 Explain how control of experimental variability can help reduce the number of experimental units needed.**	**10.210.6**
	**B7 Describe the use of power analysis to estimate the power of a proposed study.**	**10.6**

Group session 2a: Randomisation	**B8** Randomise experimental units to treatments in a completely randomised design	**10.3**
Group session 2b: Power analysis	**B9** Estimate the number of experimental units needed for a proposed experiment using power analysis	**10.6.**
Animal models and harm-benefit analysis	**A22** List the criteria used to assess the suitability of a potential non-animal or animal model for biomedical investigations.	**11.5,**
	**A23** Define fidelity and discrimination and give an example of a high-fidelity model and a high discrimination model.	**10.1**
	**A24** Describe briefly how a harm-benefit analysis would be carried out to meet legal requirements and ethical considerations.	**9.5**
Reproducibility issues from the conduct of an experiment	**C1** Define the term reproducibility in the context of animal experimentation.	**11.3**
	**C2** Define the term replication as applied to an experiment.	**11.3**
	**C3** Define the terms generalisability and translational as applied to animal experiments.	**11.3**
	**C4** List the ways in which the conduct of a study is liable to lead to poor reproducibility.	**11.3,**<br>**11.8**
	**C5** Define the terms internal validity and external validity.	**11.3**
	**C6** Explain with examples the concepts of face and construct validity of a model.	**11.3**
Group session 3 – errors in design	**C7** Pick up design mistakes in a range of experiments.	**11.3**
	**C8** Identify opportunities for concealed treatment allocation and blinded outcome assessment in different experimental examples.	**10.3, 11.3**
Standard designs Picking a suitable design	**D1** List different experimental designs suitable for hypothesis-testing experiments.	**10.7**
	**D2 Describe the concept of blocking and list the advantages and disadvantages of blocking.**	**11.3**
	**D3 Describe different blocking arrangements.**	**11.3**
	**D4 State the main feature(s) of and give an example of each of the following designs or experimental arrangements:**<br>**fully (completely) randomised**<br>**randomised block**<br>**factorial;**<br>**cross-over;**<br>split-plot	**11.3**
	**D5** List the main advantages and disadvantages or potential problems with each of these.	**11.3**

Group session 4 – designs	**D7 Identify blocks in different scenarios.**	**11.3**
	**D8 Select a suitable blocking arrangement in a proposed experiment.**	**11.3**
	**D9** Match the appropriate requirements to different types of experiments.	**11.3**
	**D10** Identify the design described in different scenarios.	**11.3**
	**D11** Select a suitable design in a proposed experiment.	**11.3**
Statistical testing: Analysis of variance	**B10** List the statistical tests commonly used in analysing preclinical research and describe briefly the use of each and the output obtained.	**11.3**
	**B11** Explain analysis of variance and the layout of an ANOVA table	**11.3**
Group session 5 – testing	**B12** Select the right test for a particular experiment.	**11.3**
	**B13** Correctly input blocks into a statistical analysis.	**11.3**
	**B14** Correctly input data from different designs into a statistical analysis.	**11.3**
	**B15** Estimate the number of experimental units for a proposed experiment using the resource equation.	**10.6**
Reproducibility issues 2	**C9** Describe the process of registration of an animal-based study and give the advantages of pre-registration.	**11.9.**
	**C10** Name one reproducibility network and indicate how a researcher can contribute to it.	**11.9**
	**C11** Define the terms p-hacking and HARKing and give reasons why these are considered unacceptable practices.	**11.3, 11.8, 11.9**
	**C12** Give examples of misleading presentation of data.	**11.9**
	**C13** List the advantages of full disclosure of data.	**11.9**
	**C14** Define raw data, processed data and meta-data.	**11.9**
	**C15** Give reasons why safe storage of data and traceability are considered important.	**11.9**
	**C16** List the components of good reporting as illustrated by the ARRIVE Guidelines.	**11.9**
